# Career trajectories of master of public health graduates from South African universities

**DOI:** 10.1186/s12960-026-01063-1

**Published:** 2026-04-01

**Authors:** Virginia Zweigenthal, Nicola Christofides, Thembelihle Dlungwane, Sogo France Matlala, Mathildah Mpata Mokgatle, Abraham Opare, Sean Mark Patrick, Nikki Schaay, Maylene Shung-King, Takalani Tshitangano, Laetitia Rispel

**Affiliations:** 1 School of Public Health, University of Cape Town, Cape Town, South Africa; 2 School of Public Health, University of the Witwatersrand, Johannesburg, South Africa; 3 Discipline of Public Health Medicine, University of KwaZulu-Natal, Durban, South Africa; 4 Department of Public Health, Sefako Makgatho Health Sciences University, Pretoria, South Africa; 5 School of Health Systems and Public Health, University of Pretoria, Pretoria, South Africa; 6 School of Public Health, University of the Western Cape, Cape Town, South Africa; 7 University of Limpopo, Polokwane, South Africa; 8 South African Research Chairs Initiative (SARChI) and School of Public Health, University of the Witwatersrand, Johannesburg, South Africa

**Keywords:** Career paths, Education, Health services, Human resources for health, Public health, Public health workforce, South Africa

## Abstract

**Background:**

The Covid-19 pandemic highlighted the need for public health professionals to be embedded in country health systems. The Master of Public Health (MPH), offered widely, is accepted as the entry degree for public health practice in low- and middle-Income countries (LMICs). The aim of the study was to address the knowledge gaps on the career trajectories of MPH graduates from South African universities.

**Methods:**

A research team from the eight South African universities that graduated MPH students between 2012 and 2016 obtained lists of alumni and invited them to participate in an on-line survey. The self-administered questionnaire elicited the demographic characteristics of MPH graduates, their educational and work background, the impact of the MPH on their subsequent work and their perspectives on the roles of MPH graduates.

**Results:**

The overall 37% response rate varied by institution. Respondents were mid-career professionals, in their mid-30s with on average, nine years work life. A sizeable proportion came from sub-Saharan Africa and they returned to their home or a neighbouring country to work. Most had been managers or patient-facing health professionals, and the MPH was the route to shift into public health roles. After MPH completion, 91% were employed in government (40%), non-governmental organisations (32%) or academic/research institutions (21%) in technical, managerial or academic roles. The MPH was a stepping stone for career advancement, and 55% of study participants changed their employers post qualification. They envisaged that MPH graduates could assume leadership positions and effectively contribute across both technical and managerial domains, including during public health emergencies.

**Conclusion:**

The MPH degree was pivotal to graduates taking on public health roles in their home countries. In view of public health workforce shortages, the study findings contribute to planning for a competent cadre to tackle pressing public health problems and their social determinants and support robust health systems development in Africa.

**Supplementary Information:**

The online version contains supplementary material available at 10.1186/s12960-026-01063-1.

## Introduction

The COVID-19 pandemic exposed critical gaps in public health systems worldwide, and underscored the need for a well-trained, adaptable, and embedded public health workforce [1]. Chronic under-investment in the health workforce and widespread shortages, the intersection of climate shocks, demographic shifts, complex disease burdens, health emergencies, and the negative impact of COVID-19, has increased the demand for health and care workers [2]. Moreover, the majority of the 2030 estimated gap of 10 million health care workers will be concentrated in low- and middle-income countries (LMICs), which face multiple disease burdens, resource-constrained health systems, and increasing migration of skilled health professionals [2].

Furthermore, the public health workforce, defined as all individuals whose primary responsibility is collaborative action for population-wide health improvement [3], remains overlooked globally [4]. This is also the case in South Africa where the primary focus of the 2030 Human Resources for Health Strategy is on the need for frontline health professionals, health leadership, management and governance [5]. Although being important priorities, investment in the public health workforce is key in sub-Saharan Africa for the development of integrated, sustainable, and comprehensive health systems to confront complex health crises [6].

The Master of Public Health (MPH), offered by many universities internationally equips graduates with the skills to identify key human health issues, analyse their determinants, and develop evidence-based interventions. Multifaceted solutions, central to public health, encompass strategies such as shaping healthy public policies, fostering supportive environments, strengthening community action, developing personal skills and re-organizing health services. Consequently, public health competencies are essential for strengthening health systems globally and advancing the Sustainable Development Goals [7].

As in many LMICs, in South Africa, the MPH degree is the primary academic pathway for individuals seeking a career in public health. Beginning in the late 1990s, the degree has been offered by several universities to create a workforce capable of improving health and healthcare in the region [8]. Students enter programmes with undergraduate degrees mostly in health, social or natural sciences.

The MPH degree is awarded by individual universities, with diversity in fields of study. There is currently no official register for MPH graduates, nor are graduates required to register with any professional body upon degree completion. As a result, the MPH functions as an academic qualification rather than a formally recognised professional credential, setting it apart from undergraduate health sciences degrees that require certification or registration within a regulated profession.

Although the MPH degree was a requirement for central hospitals’ chief executive officer appointments in some South African provinces, there has been little emphasis on integrating this growing public health workforce in national policy [9]. No requirement for public health qualifications for employment in country public health roles is also noted in South Asia [10] and Israel [11].

Several studies have highlighted the importance of research on the public health workforce in recognition of its criticality to post-pandemic health system recovery [12–14]. A 2020 labour market analysis in the United States of America (US) underscored the importance of the public health workforce, but pointed to the difficulties to define, classify, and enumerate this group due to an inconsistent definition of public health professionals, lack of licensure or certification of public health professionals, and the lack of central registries of these professionals [14]. A 2023 scoping review, examining the professionalisation of the public health workforce, found fragmented initiatives and insufficient support from health policymakers, and recommended investments in strengthening the public health workforce worldwide [13].

There remain knowledge gaps on the profile of MPH graduates, their career trajectories, how training shapes their careers and contributions to national health systems in LMICs, especially those in sub-Saharan Africa. This study begins to address these knowledge gaps by providing the first national-level profile of MPH graduates from South African universities, focussing on those who completed degrees between 2012 and 2016. The study offers insights into the role of MPH programs in strengthening the public health workforce by examining MPH graduates’ demographic, educational and work backgrounds, their career trajectories, and perspectives on potential roles of MPH graduates. The relevance of the study is both at scholarly and policy levels. The study generates new knowledge on South African MPH programs and their graduates, contributing to the discourse on the public health workforce and its professionalisation. Findings also have implications for LMIC workforce planning, curriculum development, and the broader goal of building resilient health systems capable of addressing both current and future public health challenges.

## Methods

### Study design

We conducted a cross-sectional online survey among MPH alumni from South African universities who graduated between 2012 and 2016 inclusive. This cohort enabled the research team to answer our study questions as they had five to ten years of work experience post qualification by which time they would most likely have settled into an existing or new job. Although we planned to conduct the research in 2020, delays due to the COVID-19 pandemic meant that fieldwork was undertaken in 2022 and 2023.

### Setting

We aimed to include all South African universities that offered the MPH degree. Eligibility criteria included all universities that had MPH graduates between 2012 and 2016 and who were willing to co-create and execute the study. Eight universities were included: Sefako Makgatho Health Sciences University (SMU), the University of Cape Town (UCT), University of KwaZulu-Natal (UKZN), University of Limpopo (UL), University of Pretoria (UP), University of Venda (Univen), University of the Western Cape (UWC), and the University of the Witwatersrand (Wits). Alumni from SMU and UL were merged, as they were one university, UL, during the focus period of the study. Those universities with newer or inactive programs were excluded.

### Development of the tool

The research team from the eight participating universities developed a self-administered questionnaire (SAQ) in line with the study objectives, drawing on previous questionnaires used in alumni studies in South Africa [15, 16]. The SAQ was piloted among MPH graduates who were not part of the 2012–2016 cohort. The tool (given in Appendix A), through drop-down menus, contained questions related to alumni’s educational background, their country of origin and work, their work experience prior to, during and after completing the MPH. Participants could insert other reasons for changing employers or work responsibilities if these were not included in the drop-down menus. Possible career paths for graduates in the light of a pandemic like COVID-19 were elicited through both closed and open-ended questions. Summary information regarding their demographics, country of origin, and their educational background is reported elsewhere [17].

### Data collection

Each participating university compiled a list of eligible graduates who completed the degree between 2012 and 2016 or graduated from 2012–2017, regardless of nationality or country of work. Those individuals who graduated in 2017 were included as they may have completed the requirements for the degree in 2016 but graduated in 2017.

University student records were used to identify 710 eligible alumni. Details of students’ academic exit dates and contact information varied by institution, which created obstacles in contacting graduates and completing data collection. Email addresses were obtained by institutional investigators or their proxies messaging or calling eligible alumni with mobile numbers, inviting participation. Graduates completed the SAQ using REDCap (Research Electronic Data Capture), a secure web application for managing online surveys and databases [18].

### Data storage and analysis

REDCap encrypted the SAQ data. No identifiers were requested, and the anonymised data was exported to STATA 13 for analysis. Data were cleaned and descriptive analyses were undertaken for variables derived from closed-ended questions. Summary statistics were calculated with appropriate measures of central tendency and dispersion for normal and non-normally distributed data. Differences in responses by demographic and training variables are reported, and t-tests, Mann–Whitney tests, Chi-square and Fisher exact tests were performed for hypothesis testing. Levels of significance for tests were p < 0.05 and confidence intervals calculated are at 95% (CI). In view of the uncertain career opportunities for South African employed graduates, career paths of those working in South Africa were compared to those working outside of South Africa.

### Estimating response bias

We calculated response rates as the proportion of respondents divided by graduates with valid email addresses. The response rate was suboptimal at less than 40%. As given in Appendix B, numbers of respondents ranged between 9 and 64 for universities, which mitigated against comparing programs. To determine whether a selection bias was present, demographic data (age, sex and local/international) comparing respondents to the source population of alumni was analysed. For one institution, we were unable to obtain precise age data and could only categorise alumni by older (> 40 years) or younger (< 40 years). The proportions of binary variables—sex (male or female), international (or local) students and old (> 40 years: older; younger: < 40 years old)—tested through chi-squared tests, were equivalent overall, as were their median ages for the seven institutions who had precise ages [median age (source population) = 34 years (IQR:29–41) compared to respondents = 35 (IQR: 29–42)]. As respondents were deemed to be similar to all alumni for the period, no weightings were applied [19].

### Ethical considerations

Initial Ethics Committee approval was obtained from UCT (HREC: 630/2018). Besides SMU, all participating universities were required to, and did, obtain their own institutional Ethics Committees’ approval.

Although eligible participants’ names were known to the investigators who facilitated recruitment, no identifiers were captured in the survey. Potential respondents received information sheets (see Appendix A) about the study, informing them that they were not obliged to answer all questions and could exit if they wished, without any consequences. Only those providing electronic consent were permitted to complete the questionnaire. Those wishing to be informed of the study findings directly were invited to give their email addresses, which were delinked from their responses and stored separately. After two weeks, automated reminders via emails through REDCap, were sent to those who had not responded.

## Results

Altogether 221 MPH graduates completed the survey. The overall response rate was 35.5%, with variations by institution ranging from 18.1% to 70.3%.

### Demographic characteristics

Detailed demographic and educational characteristics of respondents, and comparison with all graduates have been reported elsewhere [17]. Table 1 summarises the socio-demographic characteristics of the respondents.

**Table 1:**
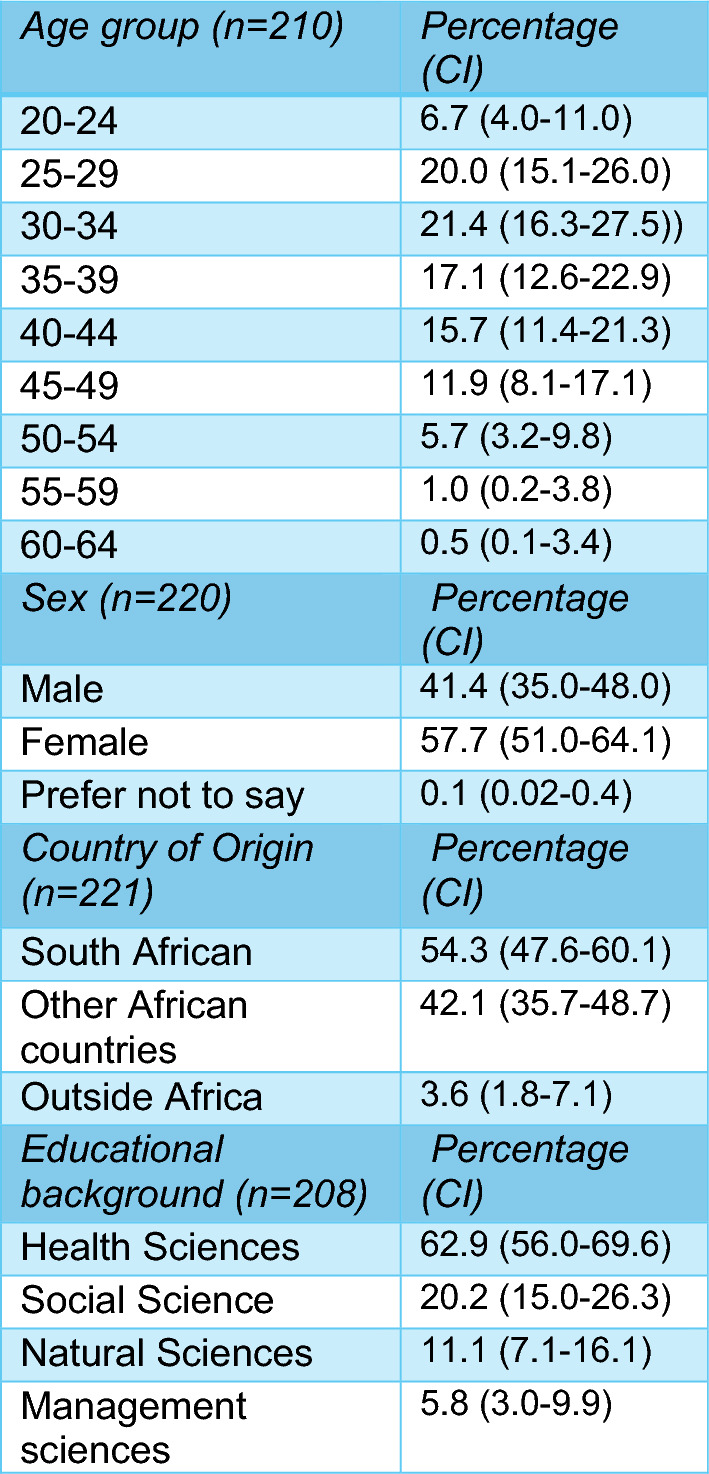
Respondents’ socio-demographic characteristics on entry to MPH

On average, the age of respondents was 35 years (median; IQR: 29–42; mean = 36.0 years; SD: 8.6 years) and was 46 years (median; IQR: 40.5–53 years; mean = 47.0 years; SD = 8.8 years) at the time of the survey. Overall, 58% of respondents were women. Most were South African (54.3%) or from other African countries (42.1%). Only 3.6% came from countries outside Africa.

### Employment trajectories

#### Prior to the MPH

Before enrolling in the MPH, respondents were employed for 9 years (median IQR: 5–16 years). As shown in Table 2, they held a range of jobs, with many (35.4%) employed in state or private sector health facilities with 79.0% being in clinical positions, followed by 21.1% who were employed in line management (managing staff delivering services to patients), or programmatic capacities in the state health sector at national, provincial or district levels or in another national government ministry. Employers of South African graduates did not significantly differ from those of non-South African graduates, except for public hospitals where a larger proportion (26.2%), of South African respondents were employed, as well as international non-governmental organisations (NGOs) and research institutes, where smaller proportions of South African respondents were employed.

**Table 2:**
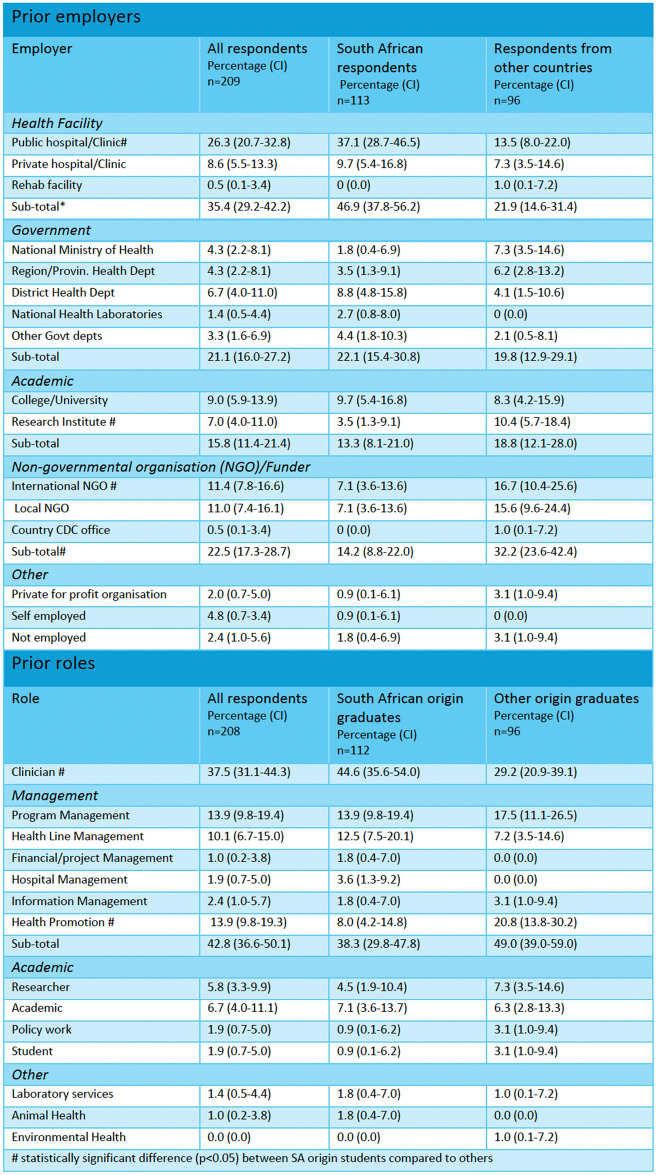
Respondents’ employers and roles prior to MPH studies, comparing South African origin to other graduates

^#^ Statistically significant difference (p < 0.05) between SA origin students compared to others

Prior to the MPH, respondents were largely managers (42.8%) followed by patient-facing-health workers (37.5%). Roles of South Africans and international respondents were equivalent except for health promotion, which was the focus of 8.0% of South African compared to 20.8% of international students (*p* = 0.01).

### Employment while studying

**Fig. 1:**
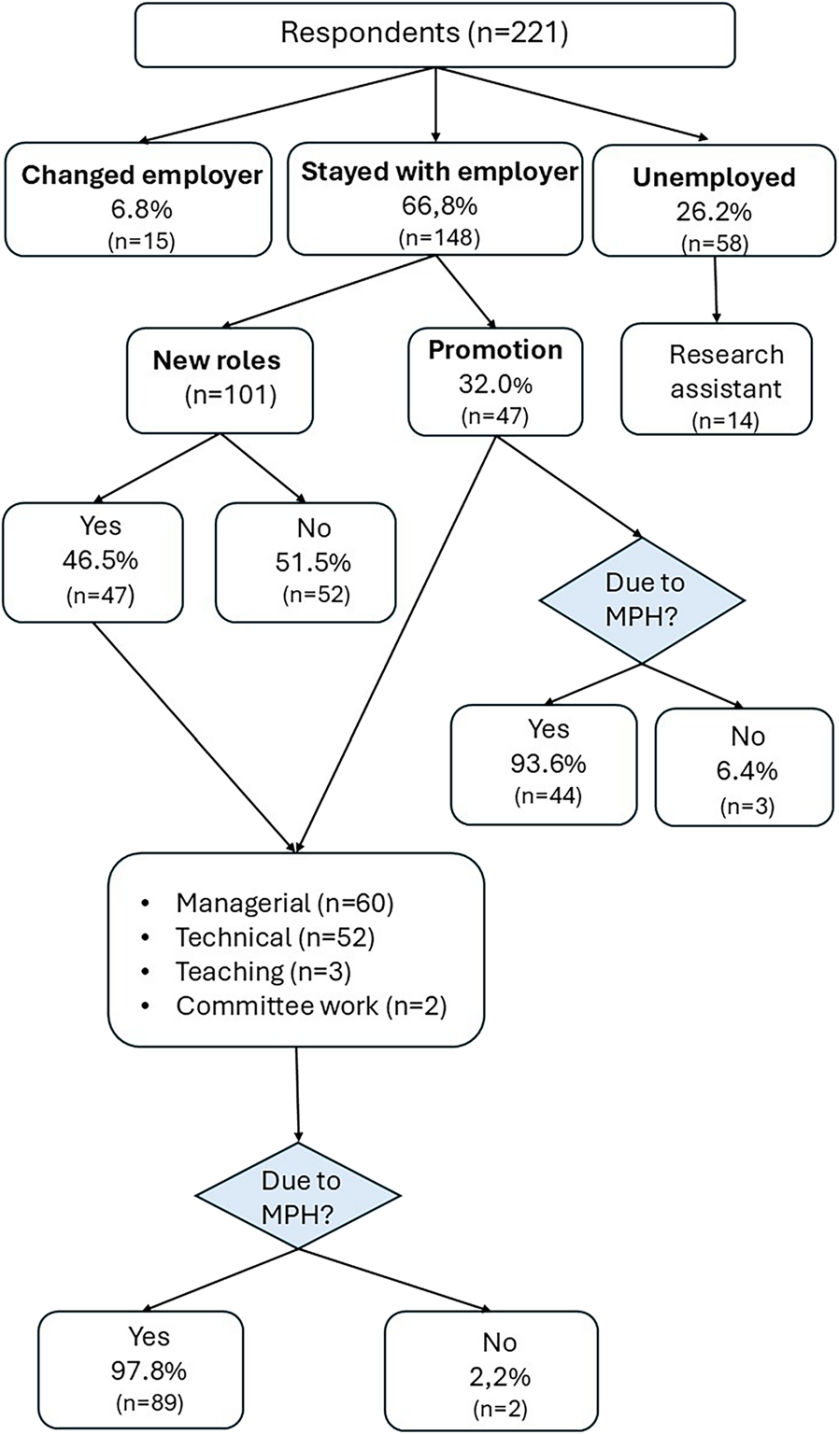
Work trajectories while completing the MPH (n = 221)

Although studying, most students (overall 66.8%; CI 60.3–73.1%) continued working for the same employers when they enrolled into the MPH, while a further 6.8% (CI 3.9–11.0%) changed employers (Fig. 1). These proportions did not differ for South African students as compared to other students (*p* = 0.84 and *p* = 0.72 respectively).

Moreover, the MPH impacted positively on respondents’ careers while they were studying. More than half of those who stayed with their employers transitioned into new roles, and nearly all (97.8%, CI 91.4–99.5%) attributed this change to completing the MPH. Many were also promoted (32.0%; CI 24.9–40.0%). Of those who were unemployed, 24.1% (CI 14.1–37.8%) worked as university research assistants while studying full-time. They were mostly international students (45.2% *p* = 0.01) compared to South Africans (13.2%).

#### Post-MPH employment

At the time of completing the survey (2022–2023) most respondents (91.3%; CI 86.7–94.4%) were employed and over half (57.8%; CI 50.8–64.1%) worked in South Africa. Respondents largely (85%) returned to their country of origin to work. Of those who relocated to another country to work (n = 33), 72.3% moved to a neighbouring African country.

Of the respondents (n = 198) who were employed at the time of the survey, 91.9% (CI 87.2–95.0%) identified that they worked in public health. Although 8.3% (CI 4.9–12.9%) were not employed at the time of the survey, 8 (47.1%; CI 2.3–7.2%) had worked in public health capacities postgraduation—in research or district health teams, with a median of 6 years (IQR: 4–6) employment.

Respondents’ career trajectories after completing the MPH are given in Table 3. They were mostly employed in the state sector (39.5%), by national or international NGOs (32.4%) and 20.9% were employed by universities or research institutes. Their roles shifted and, at the time of the survey, most graduates (26.7%) worked as program managers followed by line managers (13.3%)—managing staff delivering services to patients. The rest worked in health promotion (11.7%), research (10.0%), as academics (9.4%), policy (8.3%), and only 6.7% were clinicians.

**Table 3:**
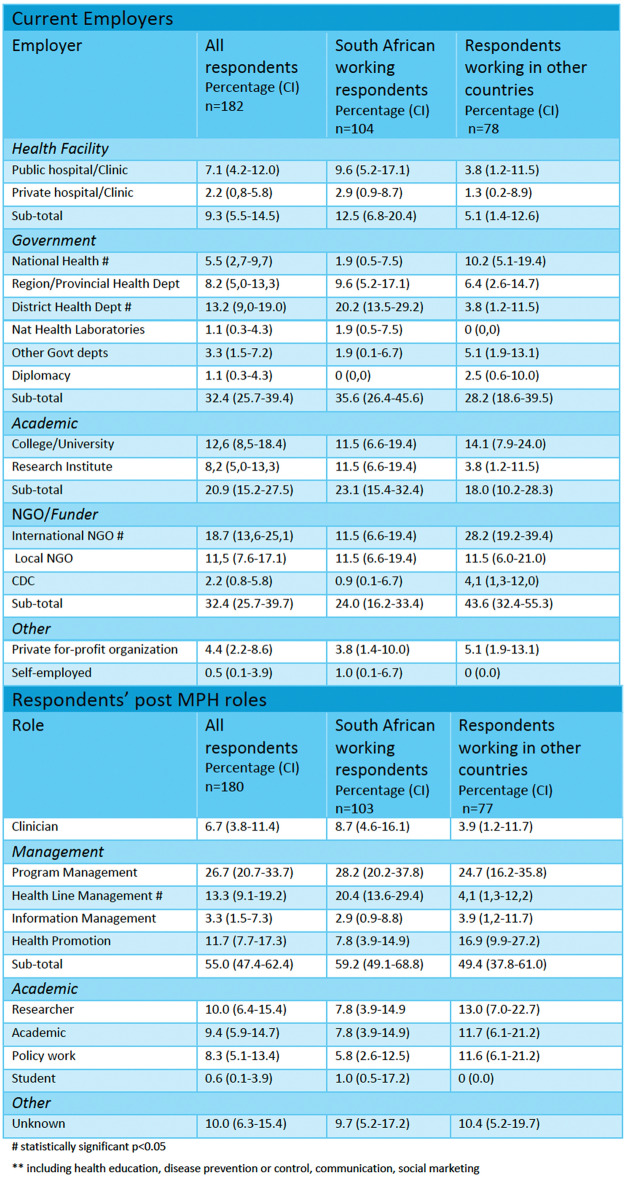
Respondents’ employers and roles post MPH studies, comparing South African employed graduates to those working elsewhere

^#^Statistically significant *p* < 0.05

^**^Including health education, disease prevention or control, communication, social marketing

Overall, there were no significant differences between alumni employed in South Africa compared to those employed outside South Africa with few exceptions. Significantly larger proportions of South African employed alumni worked in district health departments (20.2% cf. 3.8% *p* = 0.00) and became line managers (20.4% cf. 4.1% *p* = 0.00). However, smaller proportions worked at a national health level and for international NGOs (1.9% cf. 10.2% *p* = 0.00 and 11.5% cf. 28.2% *p* = 0.00 respectively). After completing the MPH, respondents’ work lives shifted. Comparing employers and roles before and after completing the MPH (in Tables 2 and 3), significantly smaller proportions of all alumni worked in hospitals (9.3% cf. 35.4%; *p* = 0.00), both public (7.1% cf. 26.3%; *p* = 0.00) and private (2.2% cf. 8.6%; *p* = 0.00), which accords with the smaller proportion who remained clinicians (37.5% cf. 6.7%; *p* = 0.00). After graduation, larger proportions worked for international NGOs (18.7% cf. 11.4%; *p* = 0.04). In addition, larger proportions of South African employed respondents worked in district health departments (20.2% cf. 8.8%; *p* = 0.02), research institutes (11.5% cf. 3.5%; *p* = 0.02), working as program managers (28.2% cf. 13.9; *p* = 0.01). Larger proportions of those employed outside of South Africa worked in policymaking (11.6% cf. 3.1%; *p* = 0.03) after completing the MPH.

**Fig. 2:**
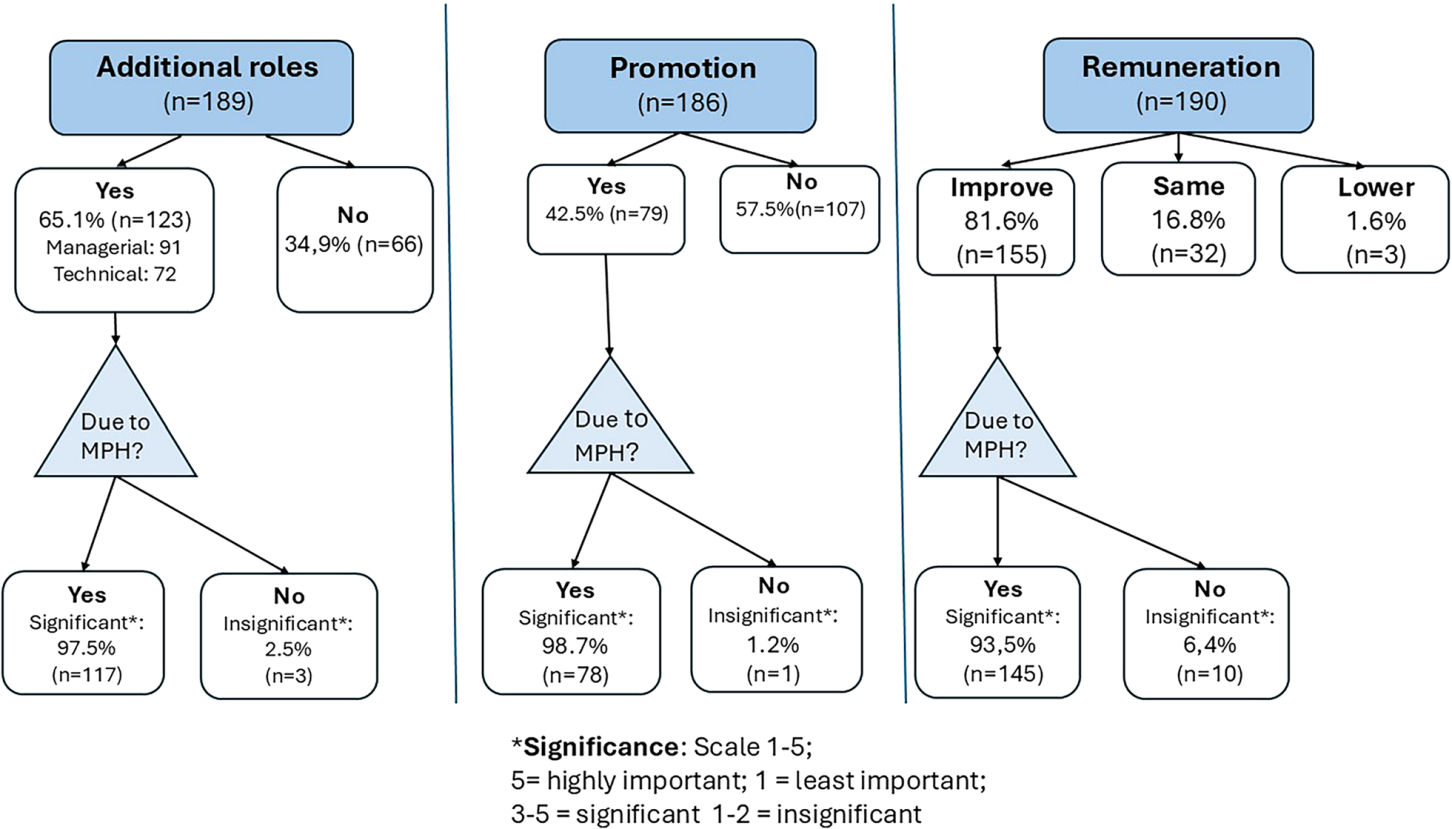
Alumni’s job trajectories after MPH completion

Employment changes are largely ascribed to the MPH (Fig. 2). Almost two-thirds (65.1%) took on additional roles, mostly managerial or technical. Furthermore, 42.5% of those who remained with employers were promoted, and most (81.6%) had salary increases. Compared to those working outside of South Africa, those working in South Africa were better remunerated after MPH completion (76.2% cf. 88.2%; *p* = 0.03) but fewer were promoted (33.9% cf. 54.5%; *p* = 0.01).

#### Reasons for job changes

**Fig. 3:**
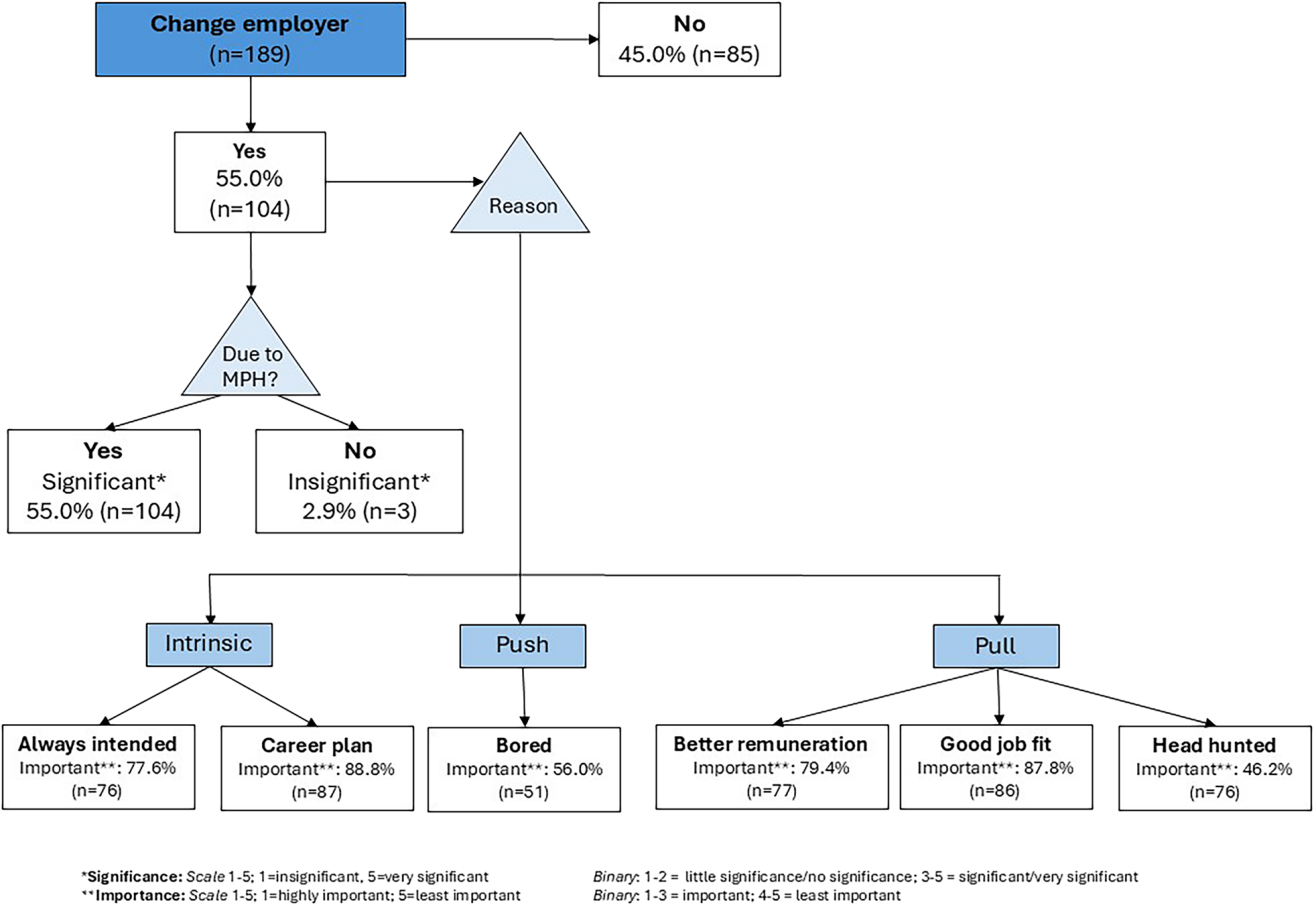
Reasons for changing employers after MPH studies

More than half (55.0%; CI 47.7–62.3%) changed employers due to their degree, for a range of reasons (Fig. 3). For 88.8%, it was part of their career plan. ‘Push’ factors include being bored by their old job (56.0%). Many were attracted by ‘pull’ factors such as an improved job fit (87.8%), better remuneration (79.4%) with some being head-hunted (46.2%). Although a higher percentage of those working abroad changed employer (60.7%), compared to those working in South Africa (48.6%), this difference was not statistically significant (*p* = 0.10). A few added that “*better conditions of work – pension*”, new opportunities such as preventive “*rather than just curative space(s)*”, the “*transfer of knowledge and skills to nurses*” and “*new challenges*” were important motivators.

#### Recommendations about MPH graduates’ roles

Over 90% of alumni agreed that employment in epidemiology, research or social and behaviour change units were excellent options for the skills of MPH graduates. In the light of the COVID-19 pandemic, volunteered recommendations were in both technical and management domains. On a technical front, they identified interpretation of data, materials development and the communication of information through the media, policy development, advising government, and evaluation of interventions. Managerially, respondents suggested that MPH graduates could be involved in coordinating functions for allocating resources, logistics planning, training as well as health facility management. These functions could be performed through graduates being employed in health services—in both public and private sectors at district, provincial or national levels—or in NGOs who are often consulted by government or by academic and research institutions. However, as one remarked, their potential “*role depend[ed] on the focus of the MPH program”*, which acknowledges the heterogeneity of MPH degrees.

## Discussion

While South African MPH programs were designed to equip professionals to tackle complex health challenges in African countries, graduates’ actual career paths have been largely unknown. This study aimed to fill this gap by collecting data from graduates of all South African universities who completed their studies between 2012 and 2016. Determining their career paths was challenging due to the lack of graduate databases, a factor also noted by Li et al. [20] in an Australian study. This was overcome to some extent in this study due to committed, insider academics who obtained alumni’s contact details.

Respondents were largely mid-career professionals who had around nine years work experience prior to their MPH studies. Consequently, most embarked on their MPHs in their 30s as was also noted in research from Australia [21] and Israel [11]. There were marginally more females (57%) than males and most were South African (54%) or from other sub-Saharan African countries, and a sizeable minority (44%) travelled to South Africa to study. They largely returned to their home country for work, signifying MPH alumni’s commitment to improving local health systems. Indeed, building public health capacity in Africa is the explicit intention of one participating university [22]. Unlike research that reports on the alarming rates of international migration of health workers from LMICs [23], graduates were retained in Africa, and the MPH was not a stepping stone for international migration with relocation to more lucrative destinations. Nonetheless, migration trends must be monitored and remediated should this shift.

As was noted in the Australian study [21], respondents had a wide range of educational backgrounds. Although small proportions had undergraduate natural and social sciences backgrounds, most (57%) worked as patient-facing clinicians in hospitals, and the MPH was a stepping stone to shift roles away from clinical work. Before embarking on the MPH, sizeable groups worked as line or project managers in governmental health facilities, in NGOs, or as academics/researchers. After graduation, only a small proportion (5.3%) returned to clinical roles, a trend consistent with other studies on MPH graduates [24, 25]. Many shifted from clinical/patient-facing roles into management, academic or research functions, similar to US public health graduates over the same time period [26]. South African programs should recognise the pivotal role of the MPH in shifting graduates’ roles towards health systems and knowledge management and hone courses to optimise learning so that graduates are ‘fit for purpose’.

The MPH not only prepared graduates for diverse roles and changed their work foci, but it also contributed to their career advancement. The vast majority of those who continued working while studying, transitioned to new public health-related roles, and a third were promoted. After graduation, similar to a US study [26], only 3.4% had not found any employment. Transitioning to public health-oriented employment had been a long-standing desire, and the additional qualification facilitated securing promotions, finding better-paying jobs that aligned with their interests. This accords with key predictors of job retention found among US government employees, being job satisfaction, opportunities for skills development and pay satisfaction [27]. These findings are consistent with international public health masters students from England [28], studies from US [29], and other multi-country [24] studies, confirming that an MPH consistently facilitates career growth and enables professionals to transition into public health managerial and technical roles, as well as research or academic work.

After completing the MPH, the range of employers were the same as before their studies. They spanned broad sectors, including government, NGOs, the private sector and universities, which mirrors findings in a 2014 Australian study [20] and international students from England [28]. Notably, despite the lack of a formal public health qualification requirement in South Africa’s health system, no South African-based respondents reported being unemployed. Half the public health South African employed respondents changed jobs after MPH completion, suggesting that the MPH qualification is valued by employers,

As is advocated by public health faculty and employers in many LMIC country settings [30], and was reported by cohorts of MD-MPH graduates in the US [31], this cohort of experienced public health graduates envisaged leadership roles for MPH graduates, believing that they could drive and function in both technical and managerial roles, including during health emergencies. They noted their skills sets were particularly useful in the interpretation of data which could inform country and health service responses in health crises, at both policy and programmatic levels. Their assessment is heartening as it accords with the intention of South African MPH programs—to equip graduates with the necessary skills to tackle complex health challenges.

### Limitations

Given the unknown career paths of MPH graduates in Africa and the uncertain career landscape in South Africa, the survey, through closed-ended questions, yielded important findings about graduates’ career trajectories, although it did not facilitate in-depth knowledge about their decision-making processes. A complementary qualitative study among MPH graduates from the 8 universities exploring these was conducted and will be reported elsewhere. The lengthy questionnaire (40–50 min), an obstacle to questionnaire completion [32], may have contributed to some incomplete submissions. To mitigate this, programming REDCap allowed participants to complete the survey in multiple sittings using a unique link.

The overall response rate, 35.5%, ranging from 18.1% to 70.3% across institutions, is slightly lower than the average 44.1% response rate found in a meta-analysis of online surveys [33], but other MPH surveys have achieved similar [24, 34] or lower [11] rates. Low response rates are a potential source of selection bias [35]. To mitigate this, we employed many recruitment strategies, including alerting alumni about the study, messaging them and phone calls, strategies used by researchers elsewhere [33]. For some, up to date contact details were not available and for others we do not know if alumni received the emails or SMSs sent to them. Despite this, if participation is not correlated with survey content, the responses of participants and non-participants should not differ significantly [35]. To check for measurable selection biases, we compared selected demographic data (sex and age) between all alumni on universities’ data bases and our respondents, factors that could be adjusted to yield more valid findings [33]. We also compared respondents to the graduate database by the binary ‘South African’ compared to ‘foreign’ alumni as the latter may be less likely respond to South African-based surveys. Respondents did not differ from the alumni database by either demographic or country of origin factors. Consequently, no weightings were applied to the data for analysis. Nonetheless, respondents may be more likely to have a positive perception of their MPH experience than non-respondents. In addition, the sample size may have introduced the wide confidence limits observed and imprecision in some findings.

## Conclusion

The recent (2024) World Health Organisation (WHO) public health workforce Roadmap highlights the importance of describing public health-trained personnel as a step in the process towards the professionalisation of a public health workforce [36]. This study aligns with this ambitious project by identifying the characteristics of MPH graduates—the cornerstone of the public health workforce—including their demographic, educational and work backgrounds, their employment and roles. It highlights the work of MPH graduates from South African universities in addressing public health needs in country health systems, which accords with the WHO's Human Resources for Health strategy [4], for the health workforce to meet global health challenges.

This study, the first known of its kind in South Africa and Africa, provides valuable insights into the career trajectories of MPH alumni from South Africa universities. It highlights the critical role of MPH training in transitioning many health professionals from clinical roles to managerial, policy, and research positions, fulfilling their career aspirations. Despite the MPH not being a formal requirement for most positions in South Africa, where most graduates worked, it clearly opened public health work opportunities, with most finding employment. Alumni largely returned to work in their home countries, demonstrating their commitment to underserved regions.

Information gleaned through the study assists teaching institutions, employers and regulators with knowledge about roles performed by public health postgraduates. This knowledge will inform discussion about the professionalisation of the public health workforce [13], an issue highlighted in global discussions following the COVID-19 pandemic.

Strengthening collaboration among South African training institutions—such as maintaining updated alumni databases to monitor and support this critical workforce; discussion on the professionalisation and registration of graduates; and, encouraging graduates to engage with public health organisations about these issues, are key steps forward. This will help advocate for embedding public health skills and professionals in national health systems in LMICs.

## Supplementary Information

Additional file1 (DOCX 236 KB)

Additional file2 (DOCX 47 KB)

Additional file3 (DOCX 16 KB)

## Data Availability

Primary data from this study is not available as consent for this from study participants was not obtained. Requests for data can be directed to virginia.zweigenthal@uct.ac.za.

## Abbreviations

CI: 95% Confidence interval
LMIC: Low- and middle-income country
MPH: Master of Public Health
MD-MPH: Medical doctor-Master of Public Health
NGO: Non-governmental organisation
REDCap: Research electronic data capture
SAQ: Self-administered questionnaire
UCT: University of Cape Town
UKZN: University of KwaZulu-Natal
UL: University of Limpopo
UP: University of Pretoria
Univen: University of Venda
UWC: University of the Western Cape
Wits: University of the Witwatersrand
WHO: World Health Organisation
