# Supplementary material for: Career trajectories of master of public health graduates from South African universities
Source: Hum Resour Health. 2026 Apr 1;24:24. doi: 10.1186/s12960-026-01063-1 (PMC13185223; doi:10.1186/s12960-026-01063-1)
Supplement: Supplementary file 1 — Additional file1 (DOCX 236 KB) [file 12960_2026_1063_MOESM1_ESM.docx]

## Contents

1. Information sheet and consent form Page 1
2. Questionnaire Page 4

## Information sheet and consent form for survey participants

## Introduction

We are a team of researchers, listed below, from different universities in South Africa that provide the Master of Public Health (MPH) degree programmes. We are conducting research on the perspectives of MPH graduates on the following:

- Reasons for embarking on an MPH,
- Perceived value and benefits,
- Career paths since MPH commencement;
- The application of their MPH learning in their work or personal lives
- The knowledge gained and skills learned as a result of the MPH
- Reflections on the role of public health during the Covid-19 disaster in South Africa.
- Their recommendation of revisions and changes to the MPH curriculum taught at South African Universities and core competencies to be included

The research involves the completion of an electronic survey, which will take about 25 minutes of your time.

## Voluntary participation

We would like you to participate in the survey, , and you are free to withdraw at any time should you wish. It will provide valuable information that could inform University and health policies in South Africa. However, your participation in this study is voluntary and there will be no negative consequences if you do not want to participate. The information obtained on MPH graduate career paths may assist the development of public health professionals’ career paths, particularly in the health systems of South Africa and other African countries as they strive to achieve the global goal of universal health coverage.

## Potential benefits and risks

There are no direct benefits, such as remuneration, for participating in the study. However, there are indirect benefits of the study. As this is the first multi-centre MPH study at South African universities, the study results could inform health workforce planning and training in South Africa, improve post-graduate selection and training programmes at the participating universities. Should you want, we will also provide you with feedback about the results.

We anticipate that there will be no risks or injury to you when participating in the study, and you will not be expected to cover any cost related to the study. Should you need data time for completing the study please let us know and we would gladly purchase data to cover this.

## Confidentiality

All the information collected will be kept strictly confidential. Study participants will all be assigned a code. These codes will only be known to members of the research team. We undertake that all information provided by you will be used only for the purpose of the study. Your identity and your individual choices will not be revealed or reported to others, or in any publication resulting from the study. Any other information that can potentially identify you in this study is being sought for follow-up purpose only and will not be used within the study. The aggregated results for each university group class and the entire group of all university MPH graduates will be published in peer reviewed journal articles.

## Ethical Approval

Ethical approval for this study has been obtained from each of the participating Universities.

## Questions

We will be happy to answer any question you have about this study. If you have any queries about the study, please contact the researcher at the institution where you trained or anyone from the list below.

| **Researcher** | **Institution** | **Contact details** |
| --- | --- | --- |
| **[name]** | **[name]** | **[email address]** |

We will be happy to answer any question you have about this study. If you have any questions about your rights as a study participant, or questions or concerns about any aspect of the study, you may contact any of the relevant individuals listed in this information sheet.

If you have any queries about the conduct of this study, you can contact the Ethics committee of the institution where you trained.

| **Institution** | **Contact name** | **Contact details** |
| --- | --- | --- |
| University of Cape Town | **[name]** | **[email address]** |
| University of KwaZulu-Natal |  |  |
| University of the Witwatersrand |  |  |
| University of the Western Cape |  |  |
| University of Venda |  |  |
| University of Limpopo |  |  |
| University of Pretoria |  |  |

**CONSENT**

| I hereby give my consent to participate in this study. | Yes | No |
| --- | --- | --- |
| I understand I am taking part freely without being coerced into doing so. | Yes | No |
| I am aware that my answers and opinions will remain confidential. | Yes | No |
| I understand that I can withdraw from the study at any time without any consequences. | Yes | No |
| I need data costs to be covered in order to participate in the study | Yes | No |
| I give consent to be contacted to participate in an interview that will explore MPH career paths, competencies and recommendations | Yes | No |
| By completing this questionnaire I confirm that I am willing to participate in this study. | Yes | No |
| I give my consent to be included in the MPH database that will be established | Yes | No |
| I understand that only the items indicated in the questionnaire will be included in the data base | Yes | No |

# Questionnaire

I confirm that I understood the consent form and
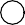
 Yes

agree to participate in this survey.
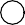
 No

## PART 1. DEMOGRAPHIC INFORMATION

- 1. Sex Female


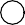

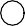

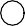


Male

Prefer not to answer

- 1. Age
  2. Which country are you from? (drop down list)
  3. Did you pursue MPH as an international or local
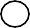
 international student student?
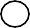
 Local student
  4. Do you have an undergraduate degree? s go to 2.3


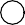

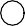


Ye

. No

## PART 2: YOUR PREVIOUS EDUCATION

- 1. Did you enter the Master of public health programme
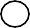
Yes
     based on a recognition of prior learning
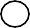
No

2.3 What was your professional education before you Bachelor of Public Health Started the MPH (Please select one option which is Business Administration Most appropriate to you) Dentistry


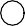

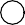

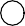

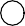

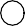

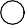

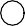

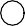

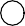

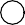

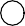

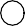

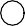

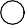

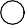

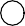

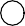


Economics

Health Management Journalism

Medical Doctor Nurse

Occupational therapy Psychology Physiotherapy Pharmacy

Speech Therapy/ Audiology Social Science

Social Work

Other

- 1. When did you complete your first degree/qualification?

## PART 3. YOUR MPH EDUCATION

3.1 Years of work experience before you started your MPH

- 1. At which University did you enroll for your MPH? * University of Cape Town

University of KwaZulu-Natal University of the Witwatersrand University of the Western Cape University of Venda


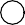

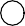

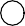

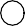

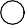

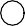

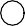

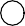


Sefako Mokgatho University of Limpopo University of Pretoria

## PART 4. YOUR PREVIOUS WORK EXPERIENCE (BEFORE MPH)

- 1. Your last place of employment BEFORE you started National Ministry of Health


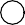

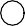

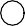

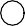

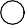

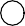

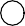

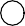

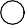

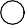

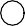

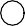

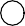


the MPH course? Regional/Provincial Health Department District Health Department

Public hospital or Clinic College/University Research Institute

International NGO Local NGO Private Hospital/Clinic

Private for-profit organization Self-employed

Not employed Other ______


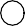

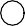


- 1. Your main area of work BEFORE starting the MPH. Clinical care/service for individual patients (Select Which one which best describes your work Health promotion (including health education,

responsibilities before MPH) * disease prevention or control, communication, social marketing, etc.)

Academic in tertiary health education institution Pure research


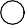

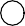


Information management

Health service (line) management Programme/project management


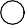

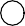


Public communication and involvement with stakeholders


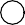

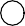


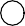
Policy process involvement Student


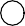
Other

## PART 5. YOUR WORK EXPERIENCE DURING THE MPH

- 1. Did you continue to work in your job during your studies?


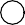
 Yes
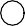
 No

- 1. During your MPH, were you promoted in your organization?


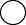
 Yes, if yes go to 5.3
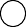
 No

Go to 5.4

Insignificant Little Moderate Significant Very significant significance significance

- 1. Please rate the contribution

of the MPH degree to the promotion

you received in your organization

5.4 Did you change jobs while you were doing the MPH?


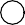
 Yes


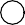
 No

- 1. Did your MPH assist you to take on any new roles while you were studying?


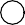
 Yes


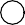
 No

- 1. I was assigned the following roles (tick all that apply)

Managerial
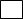


Technical
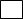


Other __


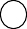

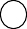

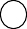

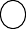

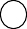
Insignificant Little Moderate Significant Very significant

5.7 Please rate the contribution of the MPH to you being assigned new roles at your workplace.

5.8 Did you get work as a research assistant at
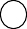
 Yes
 the university during your studies
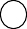
 No

(only if worked during studies = no)

## PART 6: YOUR CURRENT EMPLOYMENT

- 1. Are you currently employed?
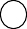
 Yes, go to 6.2 then to 6.7

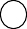
 No go to 6.3
  2. If you are currently employed, in which country?
  3. If you are currently unemployed, have you had
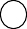
 Yes go to 6.4 any employment since completing your MPH?
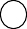
 No
  4. In which country was your most recent work?
  5. Was your most recent work public health related? Yes go to 6.6


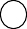

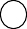


No

- 1. How many years did you work in a public health

related job since your MPH graduation

- 1.
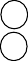
Is your current work public health related? Yes, go to 6.8

No

- 1. How many years have you been working in ? a public health related job since your MPH graduation?
  2. What best describes your CURRENT place of National Ministry of Health employment (Please select ONE option which is most Regional/Provincial Health Department appropriate to you) District Health Department


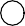

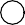

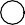

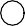

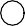

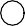

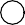

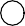

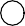

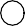

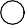

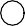

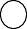


Public hospital or Clinic College/University

Research Institute International NGO

Local NGO

Private Hospital/Clinic

Private for-profit organization Self-employed

Other

- 1. Which of the following most comprehensively or Clinical care/service for individual patients best describes your work responsibilities at your Health promotion (including health education, CURRENT WORKPLACE disease prevention or control, communication,


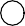

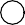


social marketing, etc)

Academic in tertiary health education institution Pu research


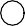

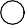


Information management
Health service (line) management Programme/project management

Public communication and involvement with stakeholders

Policy process involvement Student

Other

- 1. After your MPH were you assigned additional roles at work?

Yes, go to 6.12

No

- 1. I was assigned the following roles (tick all that apply)

Managerial

Technical

Other

Insignificant Little Moderate Significant Very significant Significance Significance

- 1. Please rate the contribution of the MPH to you being assigned additional roles
  2. After your MPH, were you promoted in your organization? Yes, go to 6.15

No

- 1. After your MPH, did your work remuneration change compared to your work

renumeration before starting the MPH?

Current renumeration is lower than before graduating from the MPH

Current renumeration is the same as before graduating from the MPH

Current renumeration is higher since graduating from the MPH

- 1. After completing the MPH, did you move to a different organization?

Yes, go to 6.18

No

Insignificant Little Moderately Significant Very Significance Significance Significant

- 1. Please rate the contribution of the MPH to your going to a different employer/organization

6.17 People move employers for a range of reasons. Rank these factors by the extent they were important for you.

From (1-highly important) to (5-least important)

No prospect for utilising my new skills

Always intended to move job after my MPH

Bored with the old job

Improved remuneration

Part of my career plan

The focus of the new organisation aligned with mine

I was head hunted by my new employer Other (Please specify: ………

PART X: WHERE MPH GRADUATES CAN BE EMPLOYED

We would like to find out your reflections on where MPH graduates can be usefully employed in the light of a pandemic like covid19. Please rate your level of agreement with the following statements.

In the light of a pandemic like covid-19, MPH graduates can be usefully employed in:

|  | 1. Strongly Disagree | 2. Disagree | 3. Neither agree nor disagree | 4. Agree | 5. Strongly agree |
| --- | --- | --- | --- | --- | --- |
| The epidemiology unit of provincial and district hospitals |  |  |  |  |  |
| Research networks located in academic, private institutions, pharmaceutical industry, NGOs etc |  |  |  |  |  |
| The National Health Laboratory services |  |  |  |  |  |
| The communication and stakeholder engagement unit in the department of health |  |  |  |  |  |
| Any other place MPH graduates can be usefully employed in the light of a pandemic like Covid-19 |  | | | | |
