# Supplementary material for: Career trajectories of master of public health graduates from South African universities
Source: Hum Resour Health. 2026 Apr 1;24:24. doi: 10.1186/s12960-026-01063-1 (PMC13185223; doi:10.1186/s12960-026-01063-1)
Supplement: Supplementary file 2 — Additional file2 (DOCX 47 KB) [file 12960_2026_1063_MOESM2_ESM.docx]

# Contents

1. MPH tracks and specialisms
2. Additional figures depicting
   1. Figure 1A Response rates by university
   2. Figure 2A Age of respondents
   3. Figure 3A Nationality of respondents
   4. Figure 4A MPH cohorts: by year start and graduation year
   5. Figure 5A Countries where respondents were working at time of survey
   6. Figure 6A Proportion of respondents who returned to home country for work
   7. Figure 7A Potential employers of MPH graduates in view of a health emergency such as Covid-19

# Categorisation of MPH tracks and specialisms

1. **Environmental and Occupational Health**
   1. Environmental health
   2. Occupational hygiene
2. **Epidemiology**
   1. Epidemiology and biostatistics
   2. Communicable and non-communicable diseases
   3. Field Epidemiology and disease control
3. **Health Systems and Policy**
   1. Health systems and policy
   2. Hospital Management
   3. Maternal and Child Health
   4. Human Resources development
   5. Health Economics
   6. Pharmaceutical public health
   7. Monitoring and Evaluation
   8. Public Health Management
4. **General**
5. **Structural and social determinants of health**
   1. Social and behavioural change communication
   2. Rural health
   3. Health promotion

# Additional Figures

**Figure 1A Numbers of respondents and response rate for the seven universities**

**Figure 2A Age of respondents at the commencement of the MPH**

**Figure 3A Respondents’ nationalities**

**Figure 4A Respondent cohort by year of MPH start and graduation**

**Figure 5A Countries where respondents were working at time of survey (n=189)**

**Figure 6A Proportion of respondents who returned to home country for work**

**Figure 10A Potential employers of MPH graduates in view of a health emergency such as Covid-19**
