# Supplementary material for: Career trajectories of master of public health graduates from South African universities
Source: Hum Resour Health. 2026 Apr 1;24:24. doi: 10.1186/s12960-026-01063-1 (PMC13185223; doi:10.1186/s12960-026-01063-1)
Supplement: Supplementary file 3 — Additional file3 (DOCX 16 KB) [file 12960_2026_1063_MOESM3_ESM.docx]

Related Manuscripts

This is the second of three articles that report on the quantitative study done among MPH graduates (2012–2016) from South African institutions. The first on graduates’ perceptions of the MPH on their leadership at work and in society has been published in Frontiers of Public Health. The third article on graduates’ self-perceived competencies is being finalised. Allied to these articles, further articles reporting on the qualitative research among MPH graduates will be forthcoming.
